# Family History of Cancer and Tobacco Exposure in Index Cases of Pancreatic Ductal Adenocarcinoma

**DOI:** 10.1155/2011/215985

**Published:** 2011-04-06

**Authors:** R. Lochan, A. K. Daly, H. L. Reeves, R. M. Charnley

**Affiliations:** ^1^Hepato-Pancreato-Biliary Unit, Department of Surgery, Freeman Hospital, Newcastle University, Newcastle upon Tyne NE7 7DN, UK; ^2^Institute of Cellular Medicine, Newcastle University, Newcastle upon Tyne NE2 4HH, UK; ^3^Northern Institute for Cancer Research, Newcastle University, Newcastle upon Tyne NE2 4HH, UK

## Abstract

*Aim*. To examine interaction between history of cancer in first-degree relatives and tobacco smoking in index patients of pancreatic adenocarcinoma. *Methods*. We carried out a case-control involving 113 patients with pancreatic adenocarcinoma and 110 controls over a 12-month period at the Freeman Hospital, Newcastle upon Tyne, UK. They were all administered a detailed tobacco exposure questionnaire and a family history questionnaire. We calculated cumulative tobacco exposure and risk for pancreas cancer. *Results*. Both smokers (OR 3.01 (95% CI: 1.73 to 5.24)) and those with a family history of malignancy (OR 1.98 (95% CI: 1.15–3.38)) were more likely to develop pancreatic cancer. Having more than one first-degree relative with cancer did not significantly further increase the risk of pancreatic cancer. Amongst pancreatic cancer cases, cumulative tobacco exposure was significantly decreased (*P* = .032) in the group of smokers (current and ex-smokers) who had a family history of malignancy [mean (SD): 30.00 (24.77) pack-years versus 44.69 (28.47) pack-years with no such history]. *Conclusions*. Individuals with a family history of malignancy are at an increased risk of pancreatic cancer. Furthermore, individuals with a family history of malignancy and who smoke appear to require a lesser degree of tobacco exposure for the development of pancreatic cancer.

## 1. Introduction

The major risk factors for pancreatic cancer are increasing age, tobacco smoking (2004; [1]), and family history of the cancer [2]. The strongest avoidable risk factor in sporadic pancreatic cancer development is tobacco smoking. Familial pancreatic cancer occurs at an earlier age, [3] is clustered in families [4, 5] and has the same poor prognosis as its sporadic counterpart. There are other inherited conditions in which pancreatic cancer occurs as part of a syndrome [6], with 5–10% of pancreatic cancer cases being associated with hereditary syndromes [7], of which familial pancreatic cancer is the most common. About 30% of sporadic pancreatic cancers are causally related to smoking. The remainder have been poorly characterised in terms of aetiology. Although a family history of cancer is known to increase the risk of pancreatic cancer, the additional effect of smoking in these patients is unknown. 

Analysis of genetic risk of cancer has shown that most nonhereditary, sporadic cancers develop in genetically predisposed individuals. This predisposition is most likely a result of several low penetrant genes rather than single-gene mutations [8, 9]. These low penetrant genes which by themselves have small relative risks, by virtue of being common in the population may have large population, attributable risks [10]. It has been observed from epidemiological studies that the first-degree relatives of sporadic cancer patients have a 2-3-fold higher risk of developing cancer at the same site and this has also been described for pancreatic cancer but in only retrospective studies [10–12]. Familial clustering observed in certain sporadic cancers without obvious Mendelian inheritance suggests that there is a genetic component in addition to environmental factors [13]. This could be explained on the basis that family members with the similar genetic background are exposed to the same environment and that this leads to the phenotypic manifestation of the disease. The interplay of environmental and genetic factors appears to play a critical role in the development of pancreatic cancer and this has been well described for its familial form [14]. On this background, it is reasonable to suppose that sporadic adenocarcinoma of pancreas, which forms the majority, is due to gene-environment interaction (GEI). These have been poorly characterised and therefore the majority of sporadic pancreatic cancers have been considered to have no identifiable cause and therefore no high-risk groups are identifiable. 

Investigation of this gene-environment interaction provides us with an opportunity to not only understand the disease better but also to stratify risks and develop strategies to improve outcome. This interindividual genetic variation modulates risk for malignancy [15] and identification of these genetic differences forms the basis of risk stratification thereby enabling targeted prevention or earlier diagnosis [16, 17]. This is especially pertinent to pancreatic cancer, as it has a particularly poor prognosis and palliation of symptoms is the most common therapy patients receive—mainly because of late diagnosis although there are other biological factors that play a role. Towards this end we have sought to investigate the relationship between these factors (tobacco smoking and a family history of malignancy) by comparing groups of patients with exposure to a known environmental risk factor for pancreatic cancer but with different genetic backgrounds.

## 2. Methods

Patients with pancreatic adenocarcinoma were prospectively identified, as part of an ongoing molecular epidemiological study. They were invited to take part in this research project which was approved by the Local Research Ethics Committee, and the clinical governance guarantor was the Newcastle upon Tyne Hospitals Foundation NHS Trust, Newcastle upon Tyne, UK. Over a period of twelve calendar months between June 2005 and May 2006, consenting individuals were administered a questionnaire which recorded, in a face-to-face interview, life-style factors including tobacco smoking habit, alcohol consumption, and occupation. A detailed family history relating to malignant disease in their first-degree relatives was also obtained directly from the patient. The World Health Organization Monitoring of Cardiovascular risks (MONICA) questionnaire was used to record detailed tobacco exposure. This enabled us to calculate cumulative tobacco exposure in individuals and to arrive at total pack-years of exposure (total pack years of smoking = (number of *cigarettes* smoked per day × number of years smoked)/20(1 pack has 20 cigarettes)). We also collected data on the mode of diagnosis of the adenocarcinoma of pancreas.

First-degree relatives (FDR) were defined as biological parents, siblings, and offspring. Individuals were considered smokers (current and ex) if they had smoked at least 100 cigarettes in their life-time and nonsmokers if they had not smoked this amount. They were considered ex-smokers if they had stopped smoking for a period of one year. Cases and controls were divided into 2 groups on the basis of a positive family history in first-degree relatives (FDR): FDR+, in whom there was history of malignancy (other than dermatological and primary brain malignancies) in first-degree relatives; and FDR−, in whom there was no such history. FDR1 denoted index cases with a single FDR with malignancy; FDR > 1 denoted those with more than one FDR with malignancy. 

We report here the interaction between tobacco exposure and a family history of malignancy in this group of patient. Continuous variables were compared by the student *t*-test and ANOVA for parametric variables and the Mann-Whitney *U* test for nonparametric variables. Correlation was tested using the Pearson's chi-square test. Directional measures were employed as necessary. Odds ratios with 95% confidence interval were calculated to quantify relative risk. SPSS version 15.0 (SPSS, Inc., Chicago IL, USA) was the software platform used for computing these tests. All continuous data are reported as mean (SD).

## 3. Results

### 3.1. Study Population

A total of 145 patients were diagnosed with pancreatic cancer in the study period, one of whom was excluded because of a diagnosis of Li-Fraumeni syndrome which is known to predispose to pancreatic cancer. Three further patients declined to enter the study leaving a total of 141 patients who agreed to take part. The mode of diagnosis of pancreatic malignancy was cytological and/or histological evidence of pancreatic ductal adenocarcinoma in 102 patients (72%) and a combination of radiological, biochemical (serially rising CA19-9), and clinical findings in 39 (28%) patients.

The controls numbering 122 were composed of patients who attended the Freeman Hospital, Newcastle upon Tyne, UK for elective hernia repair surgery (*n* = 13), cholecystectomy (*n* = 25), endoscopic treatment of bile duct stones, and/or benign biliary strictures (*n* = 9) and patients attending the anticoagulation clinic (indications included cardiac arrhythmia, prosthetic cardiac valves in-situ, following pulmonary embolism and other nonneoplastic conditions) (*n* = 75). All patients with benign biliary strictures were followed up for a median of 38 months (range 30–54) and are all currently well with no diagnosis of malignancy. Aetiology of these strictures was previous surgery in the vicinity (cholecystectomy, gastrectomy for benign disease) and previous biliary pancreatitis.

### 3.2. Cases and Controls

Of the 141 cancer patients, 113 with reliable family history were included into this study (family history data being unavailable in 21 and incomplete in 7). Of these 113 pancreatic cancer patients, 60 had a family history of a malignancy in first-degree relatives (caFDR+) whilst 53 were caFDR−. The mean (SD) age at diagnosis for pancreatic cancer cases was 65.1 (10.67) years. There was no difference (*P* = .35) in the mean (SD) age between caFDR+ and caFDR− groups (65.93 (8.90) and 62.23 (13.65) years, resp.). The overall gender ratio was 66 : 47 (m : f), (34 : 26 for caFDR+ and 32 : 21 for caFDR−).

 The controls numbered 122, of which 110 were included due to constraints of reliability or completeness of family history: controls with a positive family history of malignancy (coFDR+) = 40, controls with a negative family history of malignancy (coFDR−) = 70, controls with unavailable family history of malignancy = 5, and controls with incomplete family history of malignancy = 7. Mean (SD) age of controls was 60.07 (14.34) years. There was no significant difference between the ages of coFDR+ and coFDR− groups. The overall gender ratio was 56 : 54 (m : f) (22 : 18 for coFDR+ and 34 : 36 for coFDR−).

Table 1 summarises the demographics, smoking behaviour, and cumulative tobacco consumption, (overall consumption and stratified by FDR status) of our study population (total 223; cases 113 and controls 110).

### 3.3. Tobacco Exposure and Risk of Pancreatic Cancer

**Figure 1:**
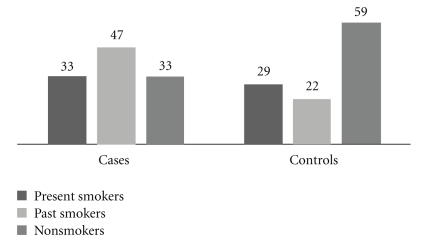
Tobacco smoking behaviour in cases and controls.

There were 80 pancreatic cancer patients who had experienced significant tobacco exposure at some point in their lives; 33 were current smokers and 47 were ex-smokers who had stopped smoking at a mean (SD) of 19.19 (14.48) years prior to diagnosis of adenocarcinoma of pancreas. The mean (SD) cumulative tobacco exposure in these 80 individuals was 36.98 (27.43) pack-years. There were 33 nonsmokers. The mean (SD) cumulative tobacco exposure in all controls who had experienced tobacco exposure (*n* = 51, current smokers = 29 and exsmokers = 22) was 37 (13.20) pack-years and this was significantly lower (*P* = .029) than that in pancreatic cancer cases. There were 59 nonsmokers amongst the control population. There was no significant difference in the number of current smokers between the cases and controls but significant differences were seen in the numbers of past smokers (Table 1 and Figure 1). 

The relative risk for an ever smoker (current and ex) for the development of pancreatic cancer is nearly 3 times that of a non-smoker (OR 3.01 (95% CI: 1.73 to 5.24)). There was no significant difference in the mean age between the cases and controls; however, there was a definite early onset of adenocarcinoma of pancreas in current smokers. A consistent early occurrence of adenocarcinoma of pancreas by about 6-7 years was seen amongst current smokers as compared to nonsmokers, which is independent of family history of cancer (Table 2). FDR status did not affect the age of onset of pancreatic cancer in our cohort (data not shown).

### 3.4. Family History of Cancer in FDRs Influencing Risk for Pancreatic Cancer

**Figure 2:**
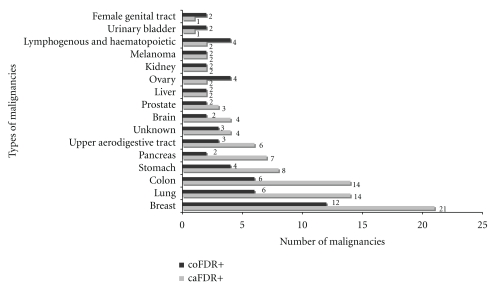
Types of malignancies in caFDR+ (*n* = 60) & coFDR+ (*n* = 40). Total number of malignancies in caFDR+ = 92 (in 60 individuals) and in coFDR+ = 58 (in 40 individuals).

A history of malignancy in FDR was present in 60 (m : f = 34 : 26) and absent in 53 (m : f = 32 : 21) cases. Amongst controls, the coFDR+ numbered 40 and coFDR− was 70. The relative risk of development of adenocarcinoma of pancreas for cases with a positive history of malignancy in FDR (caFDR+) was nearly twice that of cases with a negative history of malignancy in FDR (caFDR−) individuals (OR 1.98 (95% CI: 1.15–3.38)). This was independent of any further risk conferred by smoking. Of the 60 cases caFDR+, 36 had a single relative with cancer, 17 had 2 relatives, 6 had 3, and one had 4 relatives with cancer. In total, there were 92 malignancies in caFDR+ and 58 in the coFDR+. The different malignancies in these groups are depicted in Figure 2.

### 3.5. Interaction between Tobacco Smoking and Family History of Cancer in FDRs in Influencing Risk for Pancreatic Cancer

Most importantly amongst cases, there was a significantly decreased cumulative tobacco exposure in the caFDR+ group (*P* = .016) as compared to the caFDR− group. The mean (SD) cumulative pack-years of smoking was 30.00 (24.77) in the caFDR+ versus 44.69 (28.77) in the caFDR− group. Mean (SD) cumulative tobacco exposure in coFDR+ was 22.45 (13.18) and that in coFDR− was 17.33 (14.11). This was not statistically different (*P* = .171). There was, however, a significantly greater tobacco exposure amongst caFDR+ than their coFDR+ case counterparts (*P* = .00) (Table 1). The relative risk for adenocarcinoma of pancreas was higher in smokers in both FDR+ (OR 2.85 (95% CI: 1.24 to 6.65)) and FDR− (OR 3.18 (95% CI: 1.48 to 6.82)) groups, but the amount of tobacco exposure lower in the caFDR+.

Next we divided the cases with a family history of cancer in their FDR into 2 groups—caFDR 1 (*n* = 36): one FDR with cancer and caFDR > 1 (*n* = 24): cases with more than 1 FDR with cancer. We did not find a significant difference in the mean (SD) cumulative pack years of tobacco smoking in between these groups (FDR1: 33.70 (29.24), FDR2: 25.07 (16.68); *P* = .269).

## 4. Discussion

Following significant advances in imaging to aid in patient selection for definitive treatment and improvement in surgical technique and perioperative care, prognosis for resectable pancreatic cancer has improved appreciably. Chemotherapy has a significant role to play in selected cases [18]. However, it does appear that further significant improvement in outcome from the illness will be directly related to the ability to detect the disease early and institute prompt management. This will require identification of high-risk groups in whom targeted screening can be employed and early or precursor lesions recognized [19] and this has been demonstrated successfully in familial forms of the disease [20] and has been found to be cost-effective [21]. 

In our prospective hospital-based case-control study, we have seen that pancreatic cancer patients smoked more than our control group and an ever-smoker individual had a 3-times higher risk for the development of pancreatic cancer than a non-smoker. These are well-recognised findings. In addition, however, there were other significant results; smokers on average developed the cancer about 6-7 years earlier than nonsmokers which was independent of a family history of malignancy and has been previously described on the basis of WHO cancer mortality data and SEER cancer incidence data [22]. More importantly a family history of malignancy in first-degree relatives appeared to decrease the amount of tobacco exposure (as measured by pack-years) required for the development of pancreatic cancer. The earlier onset of the disease was however not related to FDR status.

It is accepted that familial pancreatic cancer appears to develop at an earlier age as compared to its sporadic counterpart, and tobacco exposure is the most important factor influencing the penetrance of the FPC gene [14]. Smokers in FPC [23, 24] and in hereditary pancreatic cancer syndromes, specifically hereditary pancreatitis patients [25], develop the disease about 10 years earlier, demonstrating the interaction between an inherited susceptibility to cancer and an environmental carcinogen. A recent report has described gene-environment interaction in a study of cases only, although the sample size was large [26]. We have now shown for the first time that smokers who also have a family history of cancer develop the disease at a lower level of exposure. In smokers, the disease also appears to develop earlier. This might be due to continued or faster accumulation of genotoxic mutations secondary to a variety of factors, one of which might be an inefficient DNA repair mechanism. Other genetic and environmental factors might play a role and this will need further elucidating. For example, a recent report has shown an earlier age of onset of pancreatic cancer in those who had a high BMI during their teen and younger years [27]. 

The groups of index cases and controls with and without a family history of cancer were comparable given their similar age distribution and gender distribution. We have obtained history of cancer in FDR from index cases and controls and it is known that such information is reliable and accurate especially with regard to FDRs [28]. The reliability of information obtained, however, decreases with regard to other relatives [29, 30], and we have therefore restricted our study to data on first-degree relatives. It has been suggested that, if anything there is under reporting of family history of cancer especially with regard to individuals with colorectal neoplasms [31]. Other details of the illness in the FDR such as age of onset (of the cancer in the relative) are unreliable especially in older probands and we have therefore not utilised such data in our study [29]. We have not performed genetic analysis in this group of patients to confirm that they are not familial cancers as most familial pancreatic cancers are not due to known mutations. It is likely that our patients represent sporadic malignancies due to the fact that the age distribution of the group of patients is normal and there was no difference in the mean (SD) of the age at diagnosis of the index cases in the FDR+ and FDR− groups (65.93 (10.67) and 64.57 (12.38) years). 

We have also demonstrated in this prospective group of patients, that those with a family history of cancer as evidenced by the occurrence of a malignancy in an FDR are not only at twice the risk of developing pancreatic cancer (OR 1.98 (95% CI: 1.15–3.38)) but more importantly require less of a genotoxic exposure as compared to those who do not have such a genetic vulnerability (Table 1). Just under 2/3rds of FDR+ index cases (*n* = 36; 59%) had just a single first-degree relative with malignancy. In the FDR+ group, there was a decreased tobacco exposure required for the development of adenocarcinoma of pancreas but this did not depend upon the number of relatives with malignancy, as the FDR > 1 group did not demonstrate a significantly decreased cumulative tobacco exposure. It is well accepted that a family history of cancer is a risk factor for most cancer types. With respect to adenocarcinoma of the pancreas, a recent meta-analysis of seven case-control and two cohort studies involving 6,568 pancreatic adenocarcinoma cases concluded that a family history of adenocarcinoma of the pancreas conferred double the risk (1.80 (95% CI: 1.48–2.12)) for the disease in individuals with such a history compared to those without [32]. A recent cohort study from the PanScan consortium [33] and prospective followup of participants of Cancer Prevention Study-II [34] have suggested an association between family history of various cancers especially prostate cancer and pancreatic cancer. An important additional finding from our study is confirmation that the presence of any malignancy in FDR, apart from dermatological and primary brain malignancies, appears to confer an increased risk for pancreatic adenocarcinoma. We have not performed specific FDR malignancy associated risk analyses in view of the small size of our study population. This is, however, intended for the future when a sufficiently large number of cases have been accrued.

 In the presence of a family history of malignancy (i.e., increased susceptibility), a decreased dose of an environmental carcinogen is sufficient to cause cancer (cumulative tobacco exposure in FDR+ (30 (24.77) versus FDR− (44.69 (28.47) (*P* = .00)). It is possible that the decreased tobacco dose demonstrated in the caFDR+ group is due to a genetic or other environmental factor which potentiates the genotoxic effect of tobacco-derived carcinogen by either impairing the processing of tobacco-derived carcinogen into inactive metabolites or causing the inefficient or incomplete repair of genetic damage induced by it. Genetic factors such as poor DNA repair, impaired carcinogen metabolism and environmental factors may interact in the development of tobacco-related cancers, including that of the lung, bladder and head and neck [35–38]. There is some evidence for this in pancreatic carcinogenesis too from molecular epidemiological studies: the presence of XRCC2 Arg188His polymorphism modulates risk for pancreatic cancer amongst smokers [39]; XPD gene polymorphisms—exon 10 Asp(312)Asn and exon 23 Lys(751)Gln polymorphisms—influence risk for smoking associated adenocarcinoma of the pancreas [40]; XRCC1 399Gln allele determines susceptibility to smoking induced pancreatic cancer [41]; deletion polymorphism in GSTT1 is associated with an increased risk of adenocarcinoma of the pancreas amongst Caucasians [42]. None of these studies, however, has ascertained the risk for smokers carrying these genotypes in the presence of a family history of malignancy. Our findings point to the presence of a high-risk group for adenocarcinoma of the pancreas. This cohort needs further characterisation and replication in larger population based and molecular epidemiological studies.

Identifying risk might help stratify individuals for pancreatic cancer screening but screening is not well established, the pickup rate is low and the false positive rate is relatively high. Surgery usually means a total pancreatectomy with all its potential complications. If we are able, however, to better quantify the risk, the benefits might be greater and identifying genetic and environmental factors is important. With the completion of the human genome project and advances in molecular epidemiological techniques, these low penetrant/polymorphic genes should become more frequently identified and their function understood; for example, genome-wide association studies have identified smokers with a non-O blood group as a significant high risk group for pancreas cancer as compared to nonsmokers of non-O blood group (OR 2.68 (95% CI: 2.03–3.54)) [43, 44]. Similarly identification of high-risk groups such as smokers with a positive family history of cancer could have implications for the earlier diagnosis by making screening for the disease possible leading to the prospect of long-term survival if not cure for more patients.

## 5. Summary

Smoking increases the risk for pancreatic cancer by about 3 times and current smokers develop the disease about 6-7 years earlier than nonsmokers. This risk is irrespective of a family history of any malignancy. In the presence of a family history of any malignancy, regardless of smoking, the risk for pancreatic cancer is double. In individuals with a first-degree family history of malignancy, the development of pancreatic cancer appears to occur at a lower level of cumulative tobacco exposure than in those patients without such a family history.

##  Conflict of Interests

The authors declare that they have no conflict of interests.

##  Funding

The authors are grateful to the Newcastle upon Tyne Hospitals Trustees for funding this study. The Trustees had no involvement in the design of the study; data collection, analysis or interpretation; writing of the paper; nor the decision to submit the paper for publication.

## Figures and Tables

**Table 1:** Summary of results.

	Pancreatic cancer cases	Controls	*P* value
Total	141	122	
Number included into analysis	113	110	

Male	66 (58%)	56 (51%)	ns
Female	47 (42%)	54 (49%)	ns
Mean age	65.1 (10.67)	60.07 (14.34)	ns (*t*-test)

Ever smokers	80	51	*P* = .023
Non smokers	33	59	

FDR+	60	40	*P* = .010 (chi-squared)
FDR−	53	70	

FDR+ Mean (SD) cumulative tobacco exposure in pack years	30.00 (24.77)*	22.45 (13.18)**	.229 (Mann-Whitney)

FDR− Mean (SD) cumulative tobacco exposure in pack years	44.69 (28.47)*	17.33 (14.11)**	.003 (Mann-Whitney)

Mean (SD) overall cumulative tobacco exposure in pack years	36.98 (27.43)	21.19 (22.04)	.008 (Mann-Whitney)

	*P = .016 (Mann-Whitney)	**P = .171 (Mann-Whitney)	

*Compares cumulative tobacco exposure between FDR+ and FDR− amongst pancreas cancer cases.

**Compares cumulative tobacco exposure between FDR+ and FDR− amongst controls.

**Table 2:** Age of onset of all cases of adenocarcinoma of pancreas (*n* = 113) by smoking status (Mean (SD) years).

Pancreas cancer patients grouped based on family history of malignancy status	Smoking status					
	Current smoker	Current and Ex-smoker	Ex-smoker	Non-smoker	Ex and non-smoker	ANOVA *P*
Combined caFDR+ and caFDR− (*n* = 113)	60.12 (8.18)		67.59 (10.10)	66.36 (12.17)		
	60.12 (8.18)			66.36 (12.17)		^#^.005
		64.51 (10.01)		66.36 (12.17)		*.40
	60.12 (8.18)				67.08 (10.95)	^+^.001

^#^Compares age of onset of pancreatic cancer between current and non-smokers.

*Compares age of onset of pancreatic cancer between non-smokers and combined group of current and ex-smokers.

^+^Compares age of onset of pancreatic cancer between current smokers and combined group of ex- and non-smokers.

## Explanation of Terms and Abbreviations

FDR: First-degree relatives
caFDR: First-degree relatives of cases
caFDR+: First degree relatives of cases with a history of malignancy
caFDR−: First degree relatives of cases without a history of malignancy
coFDR: First degree relatives of controls
coFDR+: First degree relatives of controls with a history of malignancy
coFDR−: First degree relatives of controls without a history of malignancy
FDR1: index cases with a single FDR with malignancy
FDR > 1: index cases with more than 1 FDR with malignancy
MONICA questionnaire: Monitoring of Cardio-vascular risks questionnaire
CI: confidence interval
SNP: Single nucleotide polymorphism
CA19-9: Carbohydrate antigen 19-9
EUS: Endoscopic ultrasound
FNA: Fine needle aspiration
ERCP: Endoscopic retrograde cholangio-pancreatography
Pack-years of cumulative smoking: = (number of *cigarettes* smoked per day × number of years smoked)/20(1 pack has 20 cigarettes).

## References

[1] Hammond EC (1964). Smoking in relation to mortality and morbidity. Findings in first thirty-four months of follow-up in a prospective study started in 1959. *Journal of the National Cancer Institute*.

[2] Li D, Xie K, Wolff R, Abbruzzese JL (2004). Pancreatic cancer. *The Lancet*.

[3] Petersen GM, De Andrade M, Goggins M (2006). Pancreatic cancer genetic epidemiology consortium. *Cancer Epidemiology Biomarkers and Prevention*.

[4] Earl J, Yan LI, Vitone LJ (2006). Evaluation of the 4q32-34 locus in European familial pancreatic cancer. *Cancer Epidemiology Biomarkers and Prevention*.

[5] Pogue-Geile KL, Chen RU, Bronner MP (2006). Palladin mutation causes familial pancreatic cancer and suggests a new cancer mechanism. *PLoS Medicine*.

[6] Habbe N, Langer P, Sina-Frey M, Bartsch DK (2006). Familial pancreatic cancer syndromes. *Endocrinology and Metabolism Clinics of North America*.

[7] Brand RE, Lynch HT (2000). Hereditary pancreatic adenocarcinoma: a clinical perspective. *Medical Clinics of North America*.

[8] Houlston RS, Peto J (2004). The search for low-penetrance cancer susceptibility alleles. *Oncogene*.

[9] Imyanitov EN, Togo AV, Hanson KP (2004). Searching for cancer-associated gene polymorphisms: promises and obstacles. *Cancer Letters*.

[10] Del Chiaro M, Zerbi A, Falconi M (2007). Cancer risk among the relatives of patients with pancreatic ductal adenocarcinoma. *Pancreatology*.

[11] Ghadirian P, Liu G, Gallinger S (2002). Risk of pancreatic cancer among individuals with a family history of cancer of the pancreas. *International Journal of Cancer*.

[12] McWilliams RR, Rabe KG, Olswold C, De Andrade M, Petersen GM (2005). Risk of malignancy in first-degree relatives of patients with pancreatic carcinoma. *Cancer*.

[13] Li FP (1990). Familial cancer syndromes and clusters. *Current Problems in Cancer*.

[14] Brentnall TA (2005). Management strategies for patients with hereditary pancreatic cancer. *Current Treatment Options in Oncology*.

[15] Lochan R, Daly AK, Reeves HL, Charnley RM (2008). Genetic susceptibility in pancreatic ductal adenocarcinoma. *British Journal of Surgery*.

[16] Singh M, Maitra A (2007). Precursor lesions of pancreatic cancer: molecular pathology and clinical implications. *Pancreatology*.

[17] Vimalachandran D, Ghaneh P, Costello E, Neoptolemos JP (2004). Genetics and prevention of pancreatic cancer. *Cancer Control*.

[18] Aung KL, Smith DB, Neoptolemos JP (2007). Adjuvant therapy for pancreatic cancer. *Expert Opinion on Pharmacotherapy*.

[19] Klapman J, Malafa MP (2008). Early detection of pancreatic cancer: why, who, and how to screen. *Cancer Control*.

[20] Canto MI, Goggins M, Yeo CJ (2004). Screening for pancreatic neoplasia in high-risk individuals: an EUS-based approach. *Clinical Gastroenterology and Hepatology*.

[21] Rulyak SJ, Kimmey MB, Veenstra DL, Brentnall TA (2003). Cost-effectiveness of pancreatic cancer screening in familial pancreatic cancer kindreds. *Gastrointestinal Endoscopy*.

[22] Raimondi S, Maisonneuve P, Löhr JM, Lowenfels AB (2007). Early onset pancreatic cancer: evidence of a major role for smoking and genetic factors. *Cancer Epidemiology Biomarkers and Prevention*.

[23] James TA, Sheldon DG, Rajput A (2004). Risk factors associated with earlier age of onset in familial pancreatic carcinoma. *Cancer*.

[24] Rulyak SJ, Lowenfels AB, Maisonneuve P, Brentnall TA (2003). Risk factors for the development of pancreatic cancer in familial pancreatic cancer kindreds. *Gastroenterology*.

[25] Lowenfels AB, Maisonneuve P, Whitcomb DC, Lerch MM, DiMagno EP (2001). Cigarette smoking as a risk factor for pancreatic cancer in patients with hereditary pancreatitis. *Journal of the American Medical Association*.

[26] Yeo TP, Hruban RH, Brody J, Brune K, Fitzgerald S, Yeo CJ (2009). Assessment of “gene-environment” interaction in cases of familial and sporadic pancreatic cancer. *Journal of Gastrointestinal Surgery*.

[27] Li D, Morris JS, Liu J (2009). Body mass index and risk, age of onset, and survival in patients with pancreatic cancer. *Journal of the American Medical Association*.

[28] Murff HJ, Spigel DR, Syngal S (2004). Does this patient have a family history of cancer? An evidence-based analysis of the accuracy of family cancer history. *Journal of the American Medical Association*.

[29] Parent MÉ, Ghadirian P, Lacroix A, Perret C (1997). The reliability of recollections of family history: implications for the medical provider. *Journal of Cancer Education*.

[30] Ziogas A, Anton-Culver H (2003). Validation of family history data in cancer family registries. *American Journal of Preventive Medicine*.

[31] Mitchell RJ, Brewster D, Campbell H (2004). Accuracy of reporting of family history of colorectal cancer. *Gut*.

[32] Permuth-Wey J, Egan KM (2009). Family history is a significant risk factor for pancreatic cancer: results from a systematic review and meta-analysis. *Familial Cancer*.

[33] Jacobs EJ, Chanock SJ, Fuchs CS (2010). A family history of cancer and risk of pancreatic cancer: a pooled analysis from the pancreatic cancer cohort consortium (PanScan). *International Journal of Cancer*.

[34] Jacobs EJ, Rodriguez C, Newton CC (2009). Family history of various cancers and pancreatic cancer mortality in a large cohort. *Cancer Causes &amp; Control*.

[35] Greer JB, Whitcomb DC (2007). Role of BRCA1 and BRCA2 mutations in pancreatic cancer. *Gut*.

[36] Wiencke JK (2002). DNA adduct burden and tobacco carcinogenesis. *Oncogene*.

[37] Barnes DE, Lindahl T (2004). Repair and genetic consequences of endogenous DNA base damage in mammalian cells. *Annual Review of Genetics*.

[38] Hung RJ, Hall J, Brennan P, Boffetta P (2005). Genetic polymorphisms in the base excision repair pathway and cancer risk: a huge review. *American Journal of Epidemiology*.

[39] Jiao LI, Hassan MM, Bondy ML (2008). XRCC2 and XRCC3 gene polymorphismand risk of pancreatic cancer. *American Journal of Gastroenterology*.

[40] Jiao LI, Hassan MM, Bondy ML, Abbruzzese JL, Evans DB, Li D (2007). The XPD AspAsn and LysGln polymorphisms, corresponding haplotype, and pancreatic cancer risk. *Cancer Letters*.

[41] Duell EJ, Holly EA, Bracci PM, Wiencke JK, Kelsey KT (2002). A population-based study of the Arg399Gln polymorphism in x-ray repair cross-complementing group 1 (XRCC1) and risk of pancreatic adenocarcinoma. *Cancer Research*.

[42] Duell EJ, Holly EA, Bracci PM, Liu M, Wiencke JK, Kelsey KT (2002). A population-based, case-control study of polymorphisms in carcinogen-metabolizing genes, smoking, and pancreatic adenocarcinoma risk. *Journal of the National Cancer Institute*.

[43] Amundadottir L, Kraft P, Stolzenberg-Solomon RZ (2009). Genome-wide association study identifies variants in the ABO locus associated with susceptibility to pancreatic cancer. *Nature Genetics*.

[44] Wolpin BM, Kraft P, Gross M (2010). Pancreatic cancer risk and ABO blood group alleles: results from the pancreatic cancer cohort consortium. *Cancer Research*.

